# Effect of lipid-based nutrient supplements on morbidity among children with stunting: secondary analysis of a randomized trial in Uganda

**DOI:** 10.1038/s41430-025-01611-3

**Published:** 2025-03-31

**Authors:** Rolland Mutumba, Joseph Mbabazi, Hannah Pesu, Jack I. Lewis, Christian Mølgaard, Christian Ritz, Mette F. Olsen, Andre Briend, Nicolette Nabukeera-Barungi, Jonathan C. Wells, Henrik Friis, Benedikte Grenov, Ezekiel Mupere

**Affiliations:** 1 Department of Paediatrics and Child Health, School of Medicine, College of Health Sciences, Makerere University, Kampala, Uganda; 2 Department of Nutrition, Exercise and Sports, University of Copenhagen, Copenhagen, Denmark; 3 The National Institute of Public Health, University of Southern Denmark, Copenhagen, Denmark; 4 Tampere Centre for Child Health Research, Tampere University, Tampere, Finland; 5 Childhood Nutrition Research Centre, Population Policy and Practice Research and Teaching Department, UCL Great Ormond Street Institute of Child Health, London, UK

**Keywords:** Nutrition disorders, Clinical trial design

## Abstract

**Background:**

Children with stunting are at risk of infections. We assessed the effect of lipid-based nutrient supplement (LNS) on morbidity in children with stunting.

**Methods:**

This was a secondary analysis of a randomized, 2×2 factorial trial among 12–59 months-old, stunted children in Uganda. Children were randomized to LNS containing milk or soy protein and whey permeate or maltodextrin, or no supplementation, for 12 weeks. The outcomes were caregiver-reported morbidity after 2, 4, 8 and 12 weeks, serum C-reactive protein (S-CRP), α_1_-acid glycoprotein (S-AGP), and phase-angle (PhA) by bioimpedance.

**Results:**

Of 750 children, mean (SD) age was 32.0 (11.7) months, 55% (*n* = 412) were male. LNS increased diarrhoea prevalence (18.1% vs 7.3%, *P* = 0.001) during the first two weeks, but not thereafter. There was no effect of LNS on cough or fever. LNS resulted in greater decline in S-AGP (−0.10 g/L, 95% CI: −0.17, −0.03, *P* = 0.003) but not S-CRP (25%, 95% CI: −11, 74, *P* = 0.193), and greater increase in PhA (0.10 degrees, 95% CI: 0.01, 0.18, *P* = 0.030), explained by greater fat-free mass. Milk compared to soy protein in LNS resulted in higher PhA (0.10 degrees, 95% CI: 0.02, 0.17, *P* = 0.013), not explained by fat-free mass.

**Conclusion:**

LNS supplementation in children with stunting had no effect on morbidity but resulted in a small reduction in sub-acute systemic inflammation. The possible effect of LNS supplementation on inflammation in stunted children requires further evaluation. (www.isrctn.com: ISRCTN13093195).

## Introduction

Globally, 38 million children with stunting are from low-income countries [1], where infectious disease morbidity and mortality are high [2]. Worldwide, 17% of deaths in children under-five-years are attributed to stunting [3], and severe stunting is associated with higher odds of mortality from diarrhoea and pneumonia [4]. Stunting is associated with impaired host defence and increased susceptibility to infections [5]. We previously reported that children with stunting from a low-income setting have micronutrient deficiencies of iron, cobalamin, and vitamin A [6]. Additionally, infections are a risk factor for stunting and micronutrient deficiencies, creating a vicious cycle [7, 8].

Children’s diets in low-income settings are predominately plant-based and often contain antinutrients which can reduce the bioavailability of essential nutrients [9, 10]. Furthermore, children in low-income settings consume less animal-source foods that contain bioavailable nutrients such as essential amino acids, vitamin A and D, zinc, iron, and vitamin B-12, that are relevant for a good immune system [11–13]. Lipid-based nutrient supplements (LNS) can be used to boost home diets by providing nutrients required for the immune system, thereby preventing and reducing severity of infections. Randomized, controlled trials (RCT) assessing the benefits of LNS on morbidity in children under-five years in low-resource settings have provided mixed results. In some RCTs, LNS supplementation reduced risk of diarrhoea, cough and fever [14–16], while others reported increased diarrhoea risk [16] or no effect on diarrhoea, cough and fever [17–19].

Phase angle (PhA), measured by bioelectrical impedance analysis (BIA), correlates with cell mass and is postulated as a marker of ‘cellular health’, reflecting the electrical integrity of the plasma membrane and the distribution of intra- and extracellular water [20, 21]. PhA may therefore act as a useful parameter in determining clinical status as positively associated with cellular integrity, and a potential marker of inflammation and oxidative stress [21, 22].

We aimed to determine the effect of LNS containing milk (MP) or soy protein and whey permeate (WP) or maltodextrin on caregiver-reported diarrhoea, cough and fever, markers of inflammation, and PhA in children with stunting.

## Methods

### Study design, setting and participants

This study was a secondary analysis of data from a trial (ISRCTN13093195) primarily testing the effect of MP and WP in LNS on growth among stunted children [23]. The trial was conducted in Jinja, eastern Uganda, and had a 2×2 factorial design with children randomized to receive one of four LNS formulations or to no intervention (control). Children aged 12–59 months within the catchment area whose length/height-for-age z-score (HAZ) was <−2, and whose caregiver provided written informed consent were enrolled. A wooden length/height measuring board-stadiometer (Weigh and Measure, Maryland, USA) was used to measure length in children <24 month and height in children ≥24 month to the nearest 0.1 cm. The median of three measurements was used. From each household, only one child was enrolled. Children with medical complications requiring hospitalization or with severe acute malnutrition (mid-upper arm circumference *<*11.5 cm or weight-for-length/height z-score *<*−3 or bipedal pitting oedema) were excluded and referred for medical care. Children with disabilities impeding measurements of length/height or their ability to eat were excluded, as were children with allergies to milk or peanut.

### Randomization and the intervention

Children were individually randomized to one of the four formulations (1:1:1:1) of LNS or the control group (4:1). Block randomization, with variable block sizes of 10 and 20, was used after stratifying by study site. R (R Core Team, 2021. R: language and environment for statistical computing, Vienna, Austria) was used to generate randomization sequences for each site by a researcher otherwise not involved. Study pharmacists at each study site received a hard copy of the randomization list in a sealed envelope and access was limited to only them. For each child enrolled, a unique identification number was sequentially allocated by a trained staff member.

The four LNS formulations were packaged in similar sachets of 100 g, each labelled with a corresponding unique three-letter code. Caregivers were blinded to the type of LNS, and outcome assessors were also blinded with regards to the child receiving the LNS intervention or not. All LNS formulations provided 530–535 kcal/sachet, contained a vitamin-mineral premix, but varied in MP and WP composition (Supplementary Table 1). Each child receiving LNS was expected to consume 1 sachet/day of LNS for twelve weeks and caregivers received refills at two-week intervals. Compliance was assessed by the number of empty sachets returned at each visit. All caregivers received nutritional counselling and transport reimbursement at each scheduled visit. In addition, caregivers of children in the control group received a bar of laundry soap.

### Measurements and follow-up

Baseline information on socio-demography, breastfeeding and morbidity was collected using interviewer-administered structured questionnaires. At inclusion, caregivers were asked whether their child had diarrhoea, cough and fever in the past 14 days followed by a clinical examination of their child. Similarly, during follow-up visits after 2, 4, 8 and 12 weeks, the caregiver was asked if the child had diarrhoea, cough or fever in the past 14 days.

At inclusion, whole blood collected in ethylenediaminetetraacetic acid (EDTA) tubes was used to test for malaria using a rapid diagnostic test (SD BIOLINE MALARIA AG PF, Abbott, USA) and to determine haemoglobin using a HemoCue device (Hb201+, Ängelholm, Sweden). In addition, human immunodeficiency virus status was determined using serial serological rapid tests (Determine HIV-1/2; Abbott, STAT-PAK; Chembio Diagnostics, Inc, and SD Bioline HIV-1/2; Standard Diagnostics, Inc).

At baseline and at week 12, serum C-reactive protein (S-CRP) and α_1_-acid glycoprotein (S-AGP) were measured at the VitMin Lab in Willstaedt, Germany, using sandwich enzyme-linked immunosorbent assay with inter-and intra-assay coefficients of variation assay between 5 and 14% [24].

PhA was automatically calculated from raw values of BIA based on resistance (R) and reactance (Xc) i.e., PhA is equal to tan^−1^(Xc/R) [21]. PhA was assessed at baseline and week 12. Bodystat 500 machines measuring at 50 kilohertz (KHz) (Bodystat, Isle of Man, United Kingdom) were used for BIA, and the mean PhA from two qualifying PhA values was used.

### Statistical analysis

As in the main trial [23], sample size considerations are based on power to detect standardized differences. In this secondary analysis, we first compared 600 children who received LNS irrespective of the milk ingredient to 150 control children and would have 80% power to detect a 0.27 standard deviation (SD) difference in the outcomes. Among the children receiving LNS, we would have 80% power to detect a 0.24 SD difference between two groups of 300 children. Participant data was double entered in EPiData (Epidata Association, Odense, Denmark) from paper case report forms and later extracted to STATA version 14 (StataCorp Ltd, Texas, USA) for analysis. Anthropometric indices were calculated using the STATA module: zscore06 [25]. We summarized categorical variables as frequency (n), and continuous variables as mean (SD) if normally distributed or median [interquartile range, IQR] if not. Changes in S-CRP, S-AGP and PhA over time from baseline to 12-weeks were analysed using a paired *t*-test.

To assess the effect of LNS, we followed an intention-to-treat analysis using available participant data. The effects of LNS (irrespective of milk ingredient) and milk ingredients (factorial design) in LNS on markers of inflammation (S-CRP & S-AGP) and PhA were determined using linear mixed models. The models were adjusted for baseline outcome value alone (model 1), and baseline value in addition to age, sex, month of inclusion and site (model 2). Logistic regression was used to analyse categorical outcomes with similar adjustments. To determine a possible contribution of fat-free mass (FFM) to the effect of LNS on PhA, we additionally adjusted for FFM at week 12. Calculation of FFM has been reported previously [23]. For right-skewed outcomes a logarithm transformation was applied before analysis, and results reported as percentages. Differences in caregiver-reported diarrhoea, cough and fever were analysed using a Pearson’s chi-square test for prevalence and Poisson regression for symptom duration. We report a Poisson regression model adjusted for age, sex, month of inclusion and site. All models were checked using residual and normal probability plots. *P* < 0.05 was considered significant. We did not adjust for multiplicity and missing data were not imputed.

### Ethical considerations

All caregivers gave oral and written informed consent for their children to participate in the study, and the study was carried out in accordance with the Helsinki Declaration. The study was approved by the School of Medicine Research and Ethics Committee of Makerere University, Kampala Uganda (#REC REF 2019-013) and the Uganda National Council of Science and Technology (SS 4927). Consultative approval was obtained from the Danish National Committee on Biomedical Research Ethics (1906848).

## Results

**Fig. 1:**
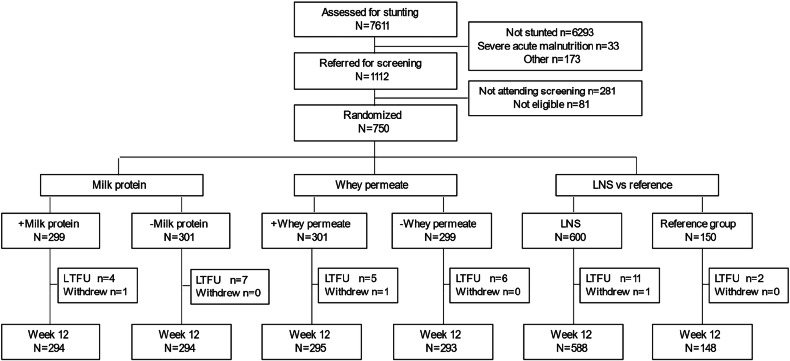
Trial profile showing the number of children assessed for stunting, screened, recruited to each intervention group, and lost to follow-up. Loss to follow-up was defined as those not returning for the 12-week visit. LNS lipid-based nutrient supplement, LTFU loss to follow-up.

Of 7611 children screened between February and September 2020, 1112 were referred for eligibility assessment, 750 were enrolled and 736 (98.1%) completed 12-week follow-up (Fig. 1). Mean (SD) age was 32.0 (11.7) months, and 55% (*n* = 412) were male. Almost half (*n* = 314) of the children had severe stunting, 65% (*n* = 479) had anaemia, 40% (*n* = 292) had malaria and 1% (*n* = 8) were HIV-seropositive. Randomization resulted in baseline equivalence (Table 1). Among children receiving LNS, 86% returned ≥80% of the sachets. Compliance did not differ between different LNS formulations (87%, 86%, 84%, and 87%, *P* = 0.87). During the trial, 10 (1.3%) children were hospitalized of whom 8 (1.3%) received LNS while 2 (1.3%) received no LNS. The main reasons for hospitalization were severe malaria and anaemia.

**Table 1:** Baseline characteristics of 750 children with stunting randomized to supplementation with lipid-based nutrient supplement (LNS) with/without milk protein (MP) and with/without whey permeate (WP) (*n* = 4×150 = 600) or no supplementation (*n* = 150)^a^.

	LNS (*n* = 600)					
	Milk vs soy protein		Whey permeate vs maltodextrin		LNS vs control	
	+MP (*n* = 299)	-MP (*n* = 301)	+WP (*n* = 301)	-WP (*n* = 299)	LNS (*n* = 600)	Control (*n* = 150)
Sociodemographic characteristics						
Age (months)	32.4 ± 11.5	31.5 ± 12.0	32.6 ± 12.1	31.2 ± 11.4	31.9 ± 11.8	32.3 ± 11.7
Sex, male	52% (156)	57% (172)	53% (159)	57% (169)	55% (328)	56% (84)
Residence, rural	44% (133)	44% (131)	43% (129)	45% (135)	44% (264)	47% (71)
Breastfed, currently	14% (42)	13% (38)	11% (32)	16% (48)	13% (80)	10% (15)
Anthropometry						
Height (cm)	81.9 ± 7.1	81.3 ± 7.5	81.9 ± 7.6	81.4 ± 7.1	81.6 ± 7.3	81.9 ± 7.5
Weight (kg)	10.7 ± 1.9	10.5 ± 2.0	10.6 ± 2.0	10.5 ± 1.9	10.6 ± 2.0	10.6 ± 2.1
Mid-upper arm circumference (cm)	14.5 ± 1.1	14.4 ± 1.2	14.5 ± 1.1	14.4 ± 1.2	14.4 ± 1.1	14.4 ± 1.3
Weight-for-height (z-score)	−0.27 ± 1.03	−0.42 ± 0.93	−0.34 ± 0.95	−0.35 ± 1.01	−0.35 ± 0.98	−0.43 ± 1.03
Height-for-age (z-score)	−3.02 ± 0.74	−3.04 ± 0.73	−3.06 ± 0.75	−2.99 ± 0.72	−3.03 ± 0.73	−2.99 ± 0.75
Morbidity in past 2 weeks^b^						
Diarrhoea	24% (73)	29% (88)	24% (71)	30 (90)	27% (161)	32% (48)
Cough	47% (141)	52% (158)	47% (141)	53% (158)	50% (299)	54% (81)
Fever	60% (178)	56 (169)	56% (169)	60% (178)	58% (347)	62% (93)
Haemoglobin <110 g/L	65% (192)	65% (192)	64% (191)	65% (193)	65% (384)	63% (95)
Positive rapid malaria test	40% (117)	37% (108)	40% (119)	36% (106)	38% (225)	45% (67)
HIV positive	2% (4)	1% (3)	2% (5)	1% (2)	1% (7)	1% (1)

^a^Data are mean ± standard deviation, or % (*n*).

^b^Caregiver-reported morbidity.

### Effect on morbidity

LNS increased the prevalence (18.1% vs 7.3%, *P* = 0.001) and the number of days (1.6 days, 95% CI: 0.9, 2.2) with diarrhoea, but only within the first two weeks (Table 2, Supplementary Table 2). The prevalence of diarrhoea declined during the trial in both the intervention and control groups. A similar overall trend of reduction in caregiver-reported cough and fever was observed, but no differences were seen between groups. WP (vs maltodextrin) tended to increase the prevalence of diarrhoea during the first two weeks (21% vs 16%, *P* = 0.10) and between 8 and 12 weeks (8% vs 3%, *P* = 0.028) (Supplementary Table 3). There was no effect of MP (vs soy) on morbidity.

**Table 2:** Prevalence of morbidity among 750 children with stunting receiving lipid-based nutrient supplements (LNS) compared to no supplement (control) at different time intervals^a^.

	Diarrhoea^b^					Cough^b^					Fever^b^				
	LNS		Control			LNS		Control			LNS		Control		
Visit	*n*	%	*n*	%	*P*	*n*	%	*n*	%	*P*	*n*	%	*n*	%	*P*
Week 2	596	18.1	150	7.3	0.001	596	35.7	150	40.0	0.33	595	33.6	150	38.0	0.31
Week 4	596	10.8	149	8.1	0.33	595	31.1	149	30.9	0.95	595	30.3	149	32.9	0.53
Week 8	587	7.0	149	6.7	0.91	588	23.8	149	22.8	0.79	590	26.1	149	24.8	0.75
Week 12	588	5.4	148	4.7	0.72	588	21.4	148	21.0	0.89	588	24.8	148	26.4	0.70

^a^Data are number of children, % prevalence and *p* values based on a Pearson’s chi-square test.

^b^Based on 14-day caregiver recall.

### Effect on markers of systemic inflammation

During the trial, children in the control group had a decline in S-AGP of 0.17 (95% CI: 0.09, 0.25) g/L (Table 3) and a marginally significant relative reduction in S-CRP (−33%, 95% CI: −56, 1). LNS, irrespective of milk ingredients, resulted in a greater decline in S-AGP (−0.10 g/L, 95% CI: −0.17, −0.03), but had no effect on S-CRP (25%, 95% CI: −11, 74). There was no effect of MP (vs soy protein), or WP (vs maltodextrin) in LNS (Table 4).

**Table 3:** Changes in markers of inflammation and phase angle among 750 children with stunting during the trial, and the effect of lipid-based nutrient supplements (LNS)^a^.

	LNS		Control		LNS vs Control			
	(*n* = 600)		(*n* = 150)		(*n* = 600 vs *n* = 150)			
	*n*		*n*		Model 1^b^	*P*	Model 2^c^	*P*
C-reactive protein^d^, mg/L								
baseline	591	1.52 [0.33; 8.58]	150	1.64 [0.37; 5.94]				
endline	585	1.20 [0.30; 5.68]	147	1.07 [0.26; 4.18]				
change (%)	576	−22 (−91, −4)	147	−33 (−56, 1)	24 (−13, 76)	0.230	25 (−11, 74)	0.193
α_1_-acid glycoprotein, g/L								
baseline	591	1.28 ± 0.51	150	1.30 ± 0.56				
endline	585	1.03 ± 0.41	147	1.14 ± 0.43				
change	576	−0.25 (−0.29, −0.21)	147	−0.17 (−0.25, −0.09)	−0.10 (−0.17, −0.03)	0.004	−0.10 (−0.17, −0.03)	0.003
Phase angle, degree								
baseline	600	3.43 ± 0.64	150	3.50 ± 0.60				
endline	588	3.68 ± 0.68	148	3.63 ± 0.66				
change	588	0.25 (0.21, 0.29)	148	0.14 (0.06, 0.22)	0.10 (0.01, 0.18)	0.037	0.10 (0.01, 0.18)	0.030

^a^Data are number of children, means ±standard deviation, median [interquartile range], mean differences or percentages (95% confidence intervals).

^b^Mean difference or percentage between LNS vs control based on linear mixed effect models adjusting for baseline value.

^c^Mean difference or percentage between LNS vs control based on linear mixed effect models adjusting for baseline value, age, sex, month of inclusion, and site.

^d^log-transformed for analysis and differences expressed as percentages.

**Table 4:** Effect of milk protein and whey permeate in lipid-based nutrient supplements (LNS) on markers of inflammation, and phase angle among 600 stunted children receiving LNS at the end of the trial^a^.

	Milk vs soy protein (*n* = 299 vs *n* = 301)			Whey permeate vs maltodextrin (*n* = 301 vs *n* = 299)			
	10^β^ or β	95% CI	*P*	10^β^ or β	95% CI	*P*	Interaction, *P*
C-reactive protein^b^, %	−10	−34, 22	0.49	12	−18, 22	0.47	0.49
α_1_-acid glycoprotein, g/L	−0.01	−0.08, 0.05	0.63	0.03	−0.03, 0.09	0.31	0.28
Phase angle, degree	0.10	0.02, 0.17	0.013	−0.01	−0.09, 0.68	0.802	0.35

^a^Data are mean difference or percentage and 95% confidence intervals (CI), based on linear mixed model adjusting for baseline value, age, sex, month of inclusion, and site; and *P* value for interaction between milk protein and whey permeate.

^b^log-transformed for analysis and percentages reported.

### Effect on phase angle

PhA increased by 0.14 (95% CI: 0.06, 0.22) degrees in the control group during the trial. LNS resulted in an additional improvement in PhA of 0.10 (95% CI: 0.01, 0.18) degrees (Table 3). Adjustment for endline FFM attenuated the magnitude of effect of LNS on PhA (0.05 degrees, 95% CI: −0.04, 0.13), indicating that part of the effect was mediated by FFM. There was no interaction between MP and WP in LNS regarding the PhA (*P* = 0.35) (Table 4). Interestingly, MP increased PhA by 0.10 (95% CI: 0.02, 0.17) degrees, and this effect was not attenuated after adjusting for endline FFM (0.10 degrees, 95% CI: 0.02, 0.18). There was no effect of WP (vs maltodextrin) on PhA.

## Discussion

LNS supplementation for 3 months transiently increased diarrhoea, had no effect on cough and fever, and resulted in a slightly greater decline in S-AGP.

Studies assessing the effect of small-quantity LNS on diarrhoea risk have reported varied results, possibly due to differences in age at recruitment and duration of LNS supplementation. A trial among South African infants found an increase in incidence of diarrhoea following 6 months supplementation with small quantity LNS [16] while other similar trials in Africa found no effect of LNS on diarrhoea [18, 19, 26, 27] or reduced diarrhoea risk [14]. Nonetheless, our findings suggest that the increase in diarrhoea was temporary as no difference in diarrhoea morbidity was observed beyond the first two weeks. The large quantity of lipid in the LNS could have caused steatorrhea, which may explain the increase in diarrhoea. Furthermore, it is possible that the increase in diarrhoea was related to the lactose in WP [28]. Due to enteric dysfunction, some children may have secondary lactose intolerance [29]. Although less likely, the iron in LNS could have contributed to a transient increase in diarrhoea since iron supplements have been shown to cause intestinal inflammation, and affect gut microbiome by reducing the abundance of commensal gut bacteria that are replaced by pathogenic bacteria [30, 31].

A positive effect of LNS supplementation on systemic inflammation was seen with S-AGP but not S-CRP. Both acute phase proteins increase during inflammation but highlight different phases of systemic inflammation. During inflammation, S-CRP rises within 4–6 h, and peaks after 24–48 h [32], whereas S-AGP takes 2–5 days to plateau and remains elevated during convalescence [33, 34]. LNS contains essential macro- and micronutrients that may help break the vicious cycle between malnutrition and inflammation [5]. Notably, systemic inflammation is associated with lower insulin-like growth factor-1 as well as growth hormone resistance, and therefore an important contributor to childhood stunting [35, 36]. Consequently, by reducing inflammation, supplementation with LNS may improve catch up linear growth in children with stunting. This is supported by findings from the primary trial, which showed that supplementation with LNS, compared to no supplementation, resulted in greater increase in insulin-like growth factor-1 [23].

LNS supplementation resulted in higher PhA, which could largely be explained by increased FFM. We have previously reported that LNS supplementation among children with stunting resulted in increased FFM and not fat mass [23]. Compared to almost no water in fat mass, FFM has a high water content making it a good conductor of electrical current hence reducing resistance which is one of the parameters in estimating the PhA [37]. Furthermore, it is possible that the lower systemic inflammation as seen in this trial favours FFM accretion and better cell function [38]. MP in LNS resulted in a higher PhA, but in this case the effect was not explained by an increase in FFM. We therefore suggest that improved ‘cellular health’ is the likely mechanism underlying the higher PhA angle values observed in the children receiving MP. A higher PhA indicates higher cell membrane integrity and better cell function [21]. The MP we used in the current study contained casein and whey proteins which, compared to soy protein, had higher quality of essential amino acids and better digestibility [39]. Furthermore, whey and casein proteins constitute bioactive peptides such as lactalbumin, lactoferrin and glycomacropetide that provide several biological functions, including immune modulation, antimicrobial and anti-inflammatory properties, and promote cell growth [40, 41].

Among the strengths of our study is the randomised design and a very low dropout rate. In addition, having BIA data enabled us to assess the possible LNS effect on PhA, which indexes FFM but not fat mass [42] and is also a promising marker of good physical health [21, 22]. As a limitation, our child morbidity data was based on reports from caregivers and therefore subject to recall bias. Nevertheless, we limited the period of recall to only 14 days. Furthermore, the families in the control group received a bar of laundry soap at each scheduled visit, and this could have reduced the diarrhoea morbidity in the control group. However, findings from large trials [43] in resource-poor settings have shown that, without efforts to support appropriate behaviour change, water, sanitation and hygiene interventions are less likely to reduce diarrhoea. Lastly, we did not directly observe children eat their LNS.

In conclusion, among children with stunting, supplementation with LNS did not have a protective effect on morbidity. However, LNS supplementation led to a small decrease in sub-acute inflammation, the importance of which is not clear and thus warrants further research. In addition, further research is needed to explore the role of MP in improving PhA independent of FFM gain.

## Supplementary information

Supplementary Tables

## Data Availability

The datasets generated during and/or analysed during the current study are not publicly available as the Uganda Act on Data Protection and Privacy and the European Act on General Data Protection Regulation do not allow for personal data to be made available to other researchers without prior written approval from relevant authorities. The data are available from the corresponding author on reasonable request.
