# Supplementary material for: Effect of lipid-based nutrient supplements on morbidity among children with stunting: secondary analysis of a randomized trial in Uganda
Source: Eur J Clin Nutr. 2025 Mar 31;79(8):756–61. doi: 10.1038/s41430-025-01611-3 (PMC12353797; doi:10.1038/s41430-025-01611-3)
Supplement: Supplementary file 1 — Supplementary Tables [file 41430_2025_1611_MOESM1_ESM.docx]

| **Supplementary Table 1.** Nutrient composition of lipid-based nutrient supplements^1^ | | | | | |
| --- | --- | --- | --- | --- | --- |
|  |  | **Milk protein^2^**  **whey permeate** | **Milk protein^2^**  **Maltodextrin** | **Soy protein^3^**  **Whey permeate** | **Soy protein^3^**  **Maltodextrin** |
| **Protein quality, DIAAS** |  |  |  |  |  |
| 6-35 months |  | 0.93 | 0.93 | 0.78 | 0.78 |
| 36+ months |  | 1.10 | 1.10 | 0.93 | 0.93 |
| **Components** | **Unit per 100g** |  |  |  |  |
| Calories | Kcal | 531 | 535 | 530 | 534 |
| Carbohydrates | g | 42 | 43 | 42 | 43 |
| Lactose | g | 15.7 | 0.4 | 15.3 | 0 |
| Proteins | g | 13.9 | 13.5 | 13.9 | 13.5 |
| Milk proteins | g | 7.15 | 6.75 | 0.40 | 0 |
| Vegetable proteins | g | 6.75 | 6.75 | 13.50 | 13.50 |
| Lipids | g | 33.7 | 33.7 | 33.7 | 33.7 |
| Linoleic acid C18:2 | g | 3.0 | 3.0 | 3.0 | 3.0 |
| Linolenic acid C18:3 | g | 0.5 | 0.5 | 0.5 | 0.5 |
| Calcium | mg | 691 | 594 | 691 | 594 |
| Copper | mg | 1.65 | 1.65 | 1.65 | 1.65 |
| Iron | mg | 12 | 12 | 12 | 12 |
| Iodine | µg | 127 | 113 | 127 | 113 |
| Magnesium | mg | 199.2 | 175.8 | 199.2 | 175.8 |
| Manganese | mg | 1.8 | 1.8 | 1.8 | 1.8 |
| Phosphorus | mg | 661 | 539 | 661 | 539 |
| Potassium | mg | 1315 | 985 | 1315 | 985 |
| Sodium | mg | 84 | 7 | 156 | 79 |
| Selenium | µg | 30 | 30 | 30 | 30 |
| Zinc | mg | 12.5 | 12.5 | 12.5 | 12.5 |
| Vitamin A | µg | 619 | 619 | 619 | 619 |
| Thiamine | mg | 1.2 | 1.1 | 1.2 | 1.1 |
| Cobalamin | µg | 3.2 | 3.0 | 3.2 | 3.0 |
| Riboflavin | mg | 3.1 | 2.8 | 2.7 | 2.4 |
| Niacin | mg | 14.9 | 14.6 | 14.9 | 14.6 |
| Pantothenic acid | mg | 5.7 | 4.5 | 5.7 | 4.5 |
| Pyridoxine | mg | 2.1 | 2.0 | 2.1 | 2.0 |
| Biotin | µg | 74.1 | 67.6 | 74.1 | 67.6 |
| Folic acid | µg | 223 | 223 | 223 | 223 |
| Vitamin C | mg | 67.9 | 67.6 | 67.9 | 67.6 |
| Vitamin D | µg | 16.9 | 16.9 | 16.9 | 16.9 |
| Vitamin E | mg | 18 | 18 | 18 | 18 |
| Vitamin K | µg | 30 | 30 | 30 | 30 |
| ^1^All lipid-based nutrient supplements were fortified with a vitamin and mineral premix.  ^2^ Milk protein isolate  ^3^ Soy protein isolate  DIAAS Digestible indispensable amino acid score. | | | | | |

| **Supplementary Table 2.** Effect of supplementation with lipid-based nutrient supplements on caregiver-reported number of days with morbidity, among 750 children with stunting at different time intervals^1,2^ (LNS, n=600 vs controls, n= 150). | | | | | | | | | | | |
| --- | --- | --- | --- | --- | --- | --- | --- | --- | --- | --- | --- |
|  | **Diarrhoea^3^** | | |  | **Cough^3^** | | |  | **Fever^3^** | | |
| Visit | B | 95% CI | P |  | B | 95% CI | P |  | B | 95% CI | P |
| Week 2 | 1.6 | 0.9, 2.2 | <0.001 |  | -0.1 | -0.4, 0.1 | 0.38 |  | -0.1 | -0.4, 0.2 | 0.51 |
| Week 4 | 0.2 | -0.7, 1.0 | 0.66 |  | 0.1 | -0.1, 0.3 | 0.58 |  | -0.1 | -0.4, 0.2 | 0.42 |
| Week 8 | 0.0 | -0.7, 0.8 | 0.91 |  | 0.2 | -0.2, 0.6 | 0.24 |  | 0.0 | -0.4, 0.4 | 0.92 |
| Week 12 | 0.2 | -0.6, 1.1 | 0.59 |  | 0.1 | -0.2, 0.5 | 0.43 |  | -0.1 | -0.4, 0.2 | 0.55 |
| ^1^ Data are regression coefficient B, 95% confidence interval, and P value.  ^2^ Analysis based on Poisson regression model adjusting for age, sex, month of inclusion, and site.  ^3^ Based on 14-day caregiver recall | | | | | | | | | | | |

| **Supplementary Table 3.** Period prevalence of diarrhoea among 600 stunted children receiving lipid-based nutrient supplements at different study visits stratified by milk ingredient ^1,2^ | | | | | | | |
| --- | --- | --- | --- | --- | --- | --- | --- |
| **visit** | **Milk protein**  **(n=299)** | **Soy protein**  **(n=301)** | **P** |  | **Whey permeate**  **(n=301)** | **Maltodextrin**  **(n=299)** | **P** |
| Week 2 | 18 (54) | 18 (54) | 1.00 |  | 21 (61) | 16 (47) | 0.10 |
| Week 4 | 12 (36) | 9 (28) | 0.29 |  | 11 (33) | 10 (31) | 0.75 |
| Week 8 | 8 (23) | 6 (18) | 0.42 |  | 6 (17) | 8 (24) | 0.26 |
| Week 12 | 5 (16) | 5 (16) | 1.00 |  | 8 (22) | 3 (10) | 0.02 |
| ^1^Data are % (n) and p values based on a Pearson’s chi-square test.  ^2^Based on 14-day caregiver recall. | | | | | | | |
